# Dried Blood Spot Sampling for Hepatitis B Virus Serology and Molecular Testing

**DOI:** 10.1371/journal.pone.0061077

**Published:** 2013-04-16

**Authors:** Sofiane Mohamed, Audrey Raimondo, Guillaume Pénaranda, Claire Camus, Denis Ouzan, Sophie Ravet, Marc Bourlière, Hacène Khiri, Patrick Dukan, Daniel Olive, Philippe Halfon

**Affiliations:** 1 Laboratoire Alphabio, Marseille, France; 2 CDL Pharma, Marseille, France; 3 Institut Arnault-Tzanck, Saint Laurent du Var, France; 4 Département d’hépato-gastroenterologie, Hôpital Saint Joseph, Marseille, France; 5 Hôpital Ambroise Paré, Marseille, France; 6 Laboratoire d’Immunologie des Tumeurs et Centre INSERM de Recherche en Cancérologie, Institut Paoli Calmettes, Marseille, France; Kaohsiung Medical University Hospital, Kaohsiung Medical University, Taiwan

## Abstract

**Background & Aims:**

Dried blood spots (DBS) on filter paper have been successfully used to diagnose and monitor several infectious diseases. The aim was to investigate the performance of DBS in hepatitis B virus (HBV) diagnosis using commercial tests in comparison to standard methods.

**Methods:**

Paired DBS and plasma samples were collected from 200 patients: 100 patients with HBsAg negative status and 100 patients with HBsAg positive status. In the latter patient, HBeAg reactivity was tested. Ten samples of anti-HBs were collected from people vaccinated against HBV. We also studied 50 patients with positive HBV DNA viral load in plasma and 10 HBV DNA negative patients. HBV genotypes and gene polymerase mutations were determined in 10 randomly selected HBV-infected patients. The DBS sample consisted of 50 µL of whole blood, i.e. a 12-mm paper card.

**Results:**

The sensitivity thresholds of HBsAg and anti-HBs antibody were 0.30±0.08 IU/mL and 18.11±6.05 IU/mL, respectively, for DBS with 98% sensitivity and 100% specificity. Sensitivity was 98% and specificity 100% for the detection of HBV DNA on a blotter, considering an HBV DNA threshold of 914.1±157.8 IU/ml. Ten patients had an HBeAg positive status in plasma, all were detected positive using DBS. HBV genotyping and mutation detection were successfully performed on DBS, with full concordance between the 10 paired DBS and plasma samples.

**Conclusion:**

This study shows DBS is a reliable alternative to plasma specimens for quantifying and detecting HBsAg, anti-HBs, HBeAg and genotyping. DBS may increase the opportunities for HBV testing and treatment follow-up in hard-to-reach individuals.

## Introduction

About one third of the world’s population has serological evidence of past or present infection with hepatitis B virus (HBV) and 350 to 400 million people are chronic HBV surface antigen (HBsAg) carriers. The spectrum of disease and natural history of chronic HBV infection range from inactive carrier status to progressive chronic hepatitis B (CHB), which may evolve to cirrhosis and hepatocellular carcinoma (HCC) [1], [2], [3]. The World Health Organization (WHO) estimated two billion people worldwide have been infected with the virus. HBV-related end stage liver disease and HCC are responsible for 0.5 to 1 million deaths per year and currently represent 5 to 10% of cases of liver transplantation [4], [5], [6], [7].. There is increased focus on prevention strategies aimed at curbing the epidemic, and therefore on screening for HBV. Early diagnosis and early treatment intervention are important. Several studies have shown that in low endemic countries some population groups have a higher prevalence of HBV infection than the general population; these include sex workers, drug users, prisoners, and immigrants from endemic countries [8], [9], [10]. However, HBV testing in these groups is limited by the poor acceptability or feasibility of venipuncture. An alternative to biological tests that require whole-blood samples obtained by venipuncture is dried blood spots (DBS) [11]. DBS can be prepared with whole blood collected from a finger stick, causing the patient less discomfort. Samples do not need to be centrifuged to separate plasma, and serum does not need to be frozen immediately after sampling. Desiccated samples can be stored for transport as nonhazardous material via postal services [12]. DBS are used for HIV-1 RNA detection by polymerase chain reaction and for viral sequencing [13], [14]. In HBV infection, DBS have been used for serology and detecting molecular biology markers such as HBV DNA, HBV core gene, anti-HBs, anti-HBc, HBsAg, hepatitis B e antigen (HBeAg) and for genotyping [15], [16], [17], [18], [19]. But most of these studies did not assess all the HBV markers on the same card and they generally did not use commercial assays.

In this study, we conducted both serological (HBs and HBe antigen, HBs antibodies) and molecular biological assays on DBS collected under different storage conditions. All tests were done using commercially available in vitro diagnostic assays. Finally, we evaluated the feasibility of DBS for genotyping and for detecting mutations in HBV polymerase gene.

## Materials and Methods

### Patients

Samples were collected from patients already attending Alphabio laboratory (Marseille, France) for HBV infection diagnosis or monitoring. In accordance with Article L1121-1 of the French Public Health guidelines, non-interventional research is not subject to a legal framework. Non-interventional research is defined as any action performed in routine without any additional procedure or unusual diagnostic or monitoring process. The patient was informed that the samples could be used for research purposes. Patients were free to refuse. The samples were used anonymously, with respect for medical confidentiality.

The study included 100 HBsAg positive plasma samples and 100 HBsAg negative plasma samples, with paired DBS. Ten samples of antibodies to HBsAg (anti-HBs) were collected from people vaccinated against hepatitis B. The presence of HBsAg and anti-HBs antibody was detected by chemiluminescent microparticle immunoassay (Abbott Diagnostics, Sligo, Ireland). Fifty samples from chronically HBV-infected patients were selected using detectable plasma HBV DNA in the automated Cobas AmpliPrep/Cobas Taqman HBV test, v2.0 (Roche Molecular System, Branchburg, NJ). The readers of results with DBS were blinded to those with plasma.

### DBS Preparation

**Figure 1:**
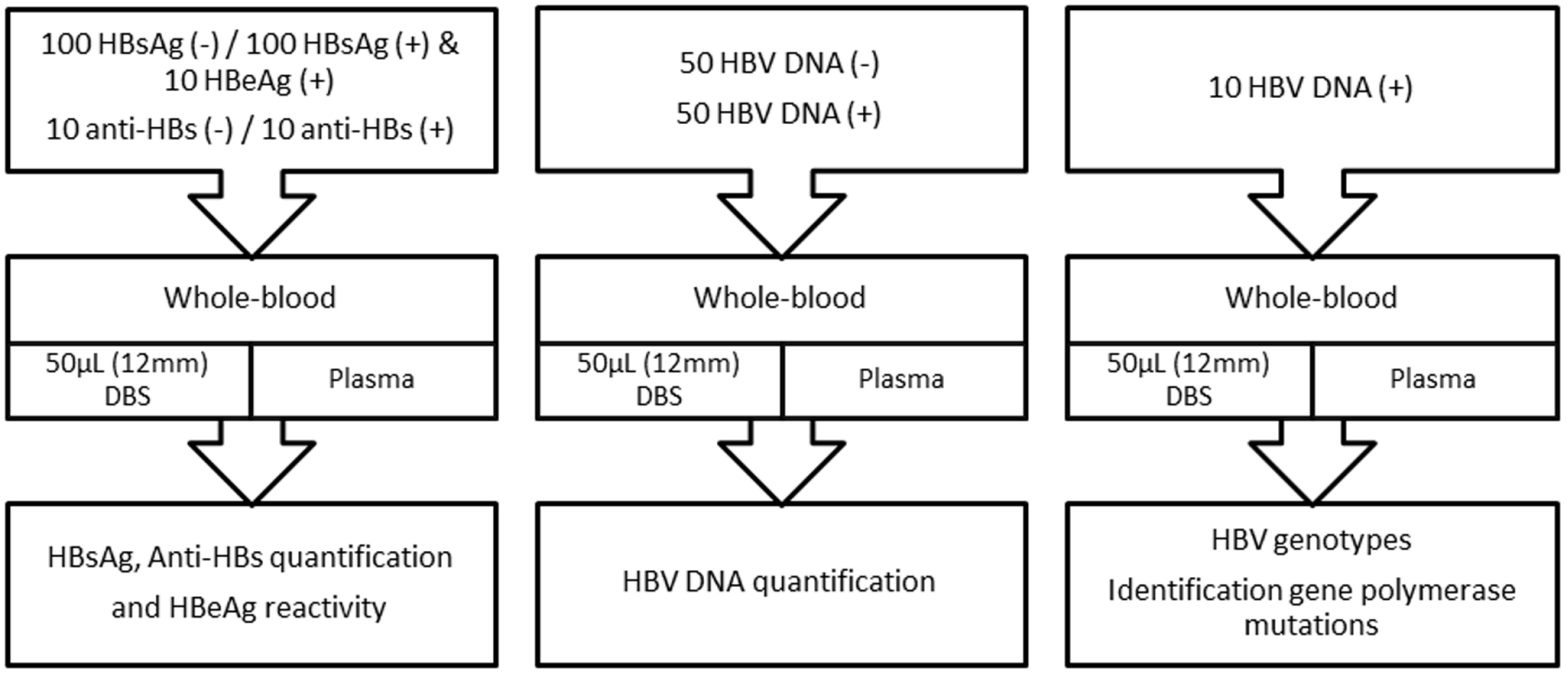
Study design. Quantification of HBsAg, anti-HBs, and HBV DNA, including characterization of HBV genotype on DBS using commercial kits. This study used DBS paired with plasma as a reference method.

One hundred and fifty microliters (three spots of 50 µL each) of venipuncture whole blood drawn in EDTA tube was absorbed onto an FTA DMPK-C card (Whatman, GE Healthcare, NJ) to completely fill three 12-mm preprinted disks on the card. The DBS cards were then dried for at least 18 h at room temperature. The blood of HBsAg and anti-HBs positive patients was used as reference material to determine the detection limit of HBsAg and anti-HBs levels. To simulate postal delay, we assessed the influence of storage at room temperature of HBsAg, anti-HBs, and HBV DNA on DBS at four time points (1 day, 3 days, 7 days, and 14 days) before quantification. Preparation of DBS samples involved dilution of HBsAg and HBV DNA from the initial EDTA blood according to the amount of plasma fixed onto the filter paper. Because the hematocrit may not always be available, we did hematocrit analysis for each patient in order to calculate the real volume of plasma in DBS. All tubes were treated less than 5 h after venipuncture. The study design is presented in **Figure 1**.

### DBS HBsAg, Anti-HBs Quantification and HBeAg Detection

A 12-mm disc was punched from the DBS cards. The disc was suspended in a 1.5-mL Eppendorff microtube with 450 µL of phosphate-buffered saline (PBS) and incubated at room temperature with continuous agitation. The disc was removed and the tube was centrifuged at 10,000 g during 10 min. We used 100µL of DBS eluate to quantify HBsAg and anti-HBs by immunoassay. The threshold values were established using the manufacturer’s instruction for HBsAg and anti-HBs plasma; they correspond to 0.05 IU/mL and 2 IU/mL, respectively. The limit of detection of HBsAg and anti-HBs for DBS was determined in 10 positive patients in duplicate with several concentrations of HBsAg and anti-HBs. One hundred microliters of DBS was also used to evaluate HBeAg reactivity by immunoassay in 100 HBsAg patients’ positives.

### HBV DNA Quantification

Elution from DBS was performed using 12-mm preprinted discs. The DBS disc was suspended in a 1.5 mL Eppendorff microtube with 700 µL of purification reagent (FTA, GE Healthcare, UK) to wash DBS during 5 min. This step was repeated two times. DBS was eluted with 700 µL of PBS and incubated at 95°C during 45 min with continuous agitation**.** We used 650 µL of eluate to detect and quantify HBV DNA by polymerase chain reaction. The threshold value was established using the manufacturer’s instruction for HBV DNA; it corresponded to <20 IU/mL. To determine HBV-DNA limit of detection, samples were collected from negative patients and were spiked with HBV plasmid (positive control, C.V.2.0, Cobas® Taqman HBV test V2.0, Roche Diagnostics, Mannheim, Germany) in duplicate at several concentrations.

### Genotyping by Sequencing

Fifty microliters of venipuncture whole blood was absorbed onto a DBS card, forming a 12-mm. The disc was suspended in a 1.5 mL Eppendorff microtube with 500 µL of purification reagent. This step was done two times. DBS was eluted with 400 µL of PBS and incubated at 95°C during 45 min with continuous agitation. After centrifugation (10 seconds at 13,000 g), 200 µL of eluted DBS was collected and DNA was extracted using NucleoSpin Blood kit (Macherey-Nagel, Germany). Genotyping was performed using the Trugene HBV Genotyping Kit Version 1.0 (Siemens Diagnostics, Tarrytown, NY) according to the manufacturer’s instructions. This assay combines the CLIP sequencing technology with automatic analysis. The resulting sequences were used to identify the HBV genotypes and the drug-resistant mutants.

### Statistical Analysis

The nonparametric Wilcoxon paired-sample test was used to compare positive HBsAg, anti-HBs, and HBV DNA samples from DBS exposed at room temperature for 1 to 14 days. Spearman’s non-parametric rank correlation coefficient and Bland-Altman [20] data plotting were used to measure relationship between DBS and plasma samples. Significance was set at *P*<0.05. Statistical analyses were performed using SAS statistical software (version 9.1.3, SAS Institute, Cary, NC).

## Results

### Sensitivity and Specificity of HBsAg and Anti-HBs Testing on DBS

**Figure 2:**
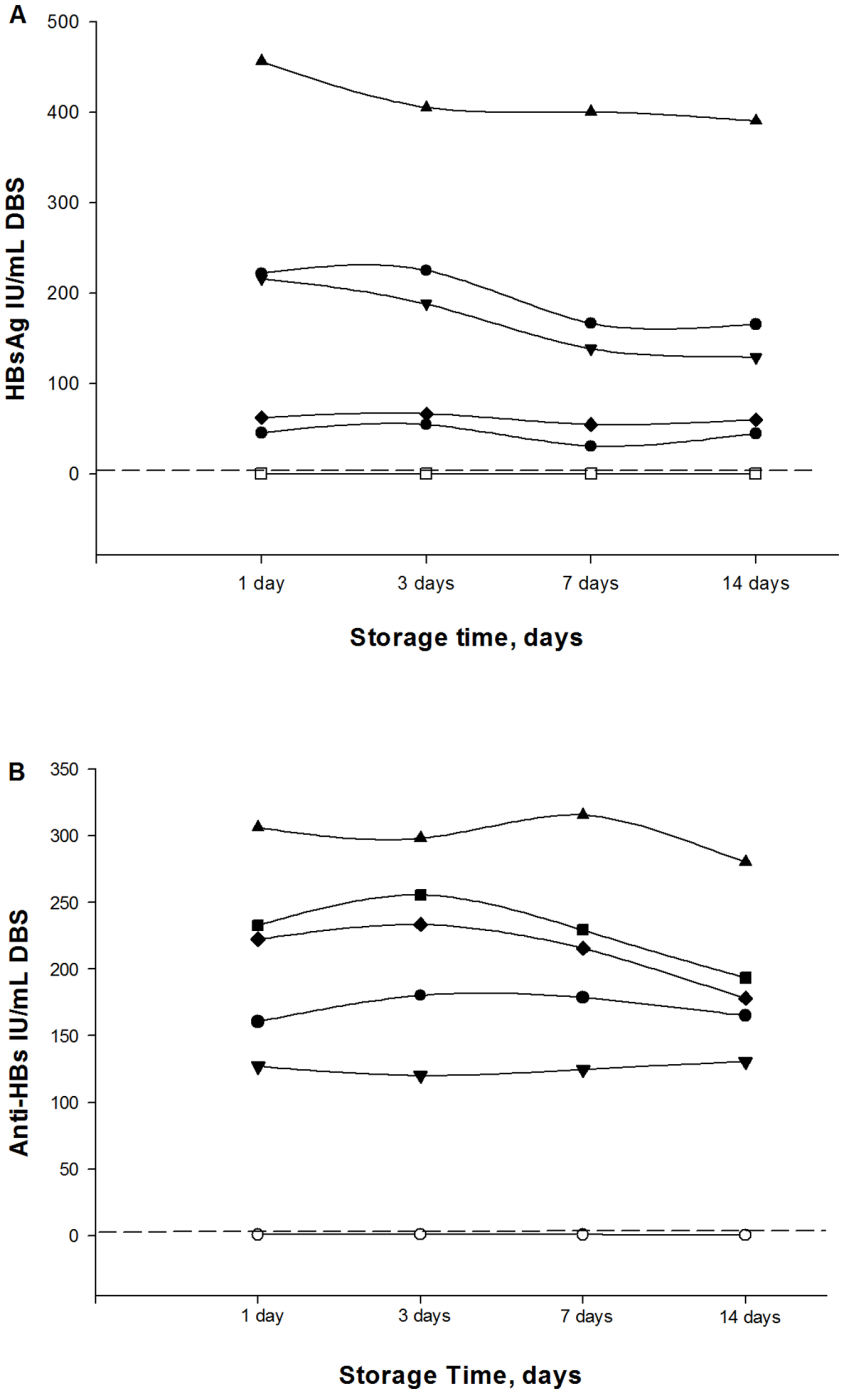
Evaluation of DBS storage relative to titer in HBsAg and anti-HBs tested by immunoassay. DBS samples were tested after storage at room temperature during 1, 3, 7, and 14 days. Each line represents 5 positive samples (black symbols) and 3 negative samples (white symbols). The dotted line represents the positive cutoff value for each parameter. (A) Evaluation of DBS storage relative to titer in HBsAg (IU/mL). (B) Evaluation of DBS storage relative to titer in anti-HBs (IU/mL).

Serial dilutions of 10 DBS and plasma samples positive for HBsAg and anti-HBs with high levels were diluted in negative donor blood and were run in duplicate to investigate the assay’s capacity to detect low levels of HBsAg and anti-HBs. The limit of HBsAg detection in DBS was 0.30±0.81 IU/mL. The lower limit of detection of anti-HBs in DBS was 18.11±6.05 IU/mL (Table 1). HBsAg and anti-HBs levels in 5 positives patients and 3 negatives patients were tested after different storage times. DBS were exposed at room temperature for 1 to 14 days and tested sequentially. In HBsAg and anti-HBs positive samples, no significant variation was observed in the titer (IU/mL) between days 1 and 14 (**Figure 2** **A and B**).

**Table 1:** Limit of detection in plasma and DBS.

Parameters	LOD Plasma IU/mL	LOD DBS IU/mL ± SD
**HBAg**	0.05	0.3±0.81
**Anti-HBs**	2	18.11±6.05
**HBV DNA**	20	914.1±157.77

LOD: limit of detection; SD: standard deviation.

### Quantification of HBsAg and HBeAg Status with DBS

**Figure 3:**
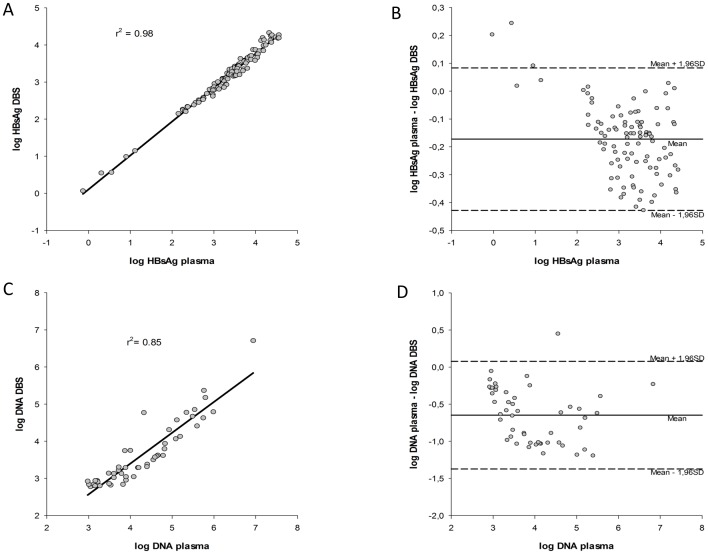
Regression analysis and Bland-Altman representation of titer differences in DBS and plasma of HBsAg levels and HBV DNA levels. (A) Linear regression analysis of HBsAg levels for the 100 matched DBS-plasma pairs analyzed by immunoassay. (B) Bland-Altman analysis was used to determine the agreement between the HBsAg titer by plotting the differences between the two samples against the averages of the two techniques. (C) Linear regression analysis of HBV DNA levels for the 50 matched DBS-plasma pairs analyzed by real-time PCR quantitative assay. (D) Bland-Altman analysis of HBV DNA.

HBsAg values obtained from DBS were compared with values from plasma. HBsAg was detected in 100% (100/100) of DBS samples with a range of 0.73 to 35771 IU/mL in plasma. A good correlation was found between HBsAg of DBS and plasma (r^2^ = 0.98; P<0.0001 (**Figure 3A**). Bland-Altman agreement analysis revealed that DBS specimens were comparable to plasma specimens for measuring HBsAg values in all but three specimens, which did not meet the 95% limits of agreement. Notably, the mean difference between plasma and DBS specimens was −0.17 log IU/mL, within the limits of agreement (mean difference ±2 SD) (**Figure 3B**). Ten patients had an HBeAg positive status in plasma, all were detected positive using DBS.

### Sensitivity and Specificity of HBV DNA Testing on DBS

**Figure 4:**
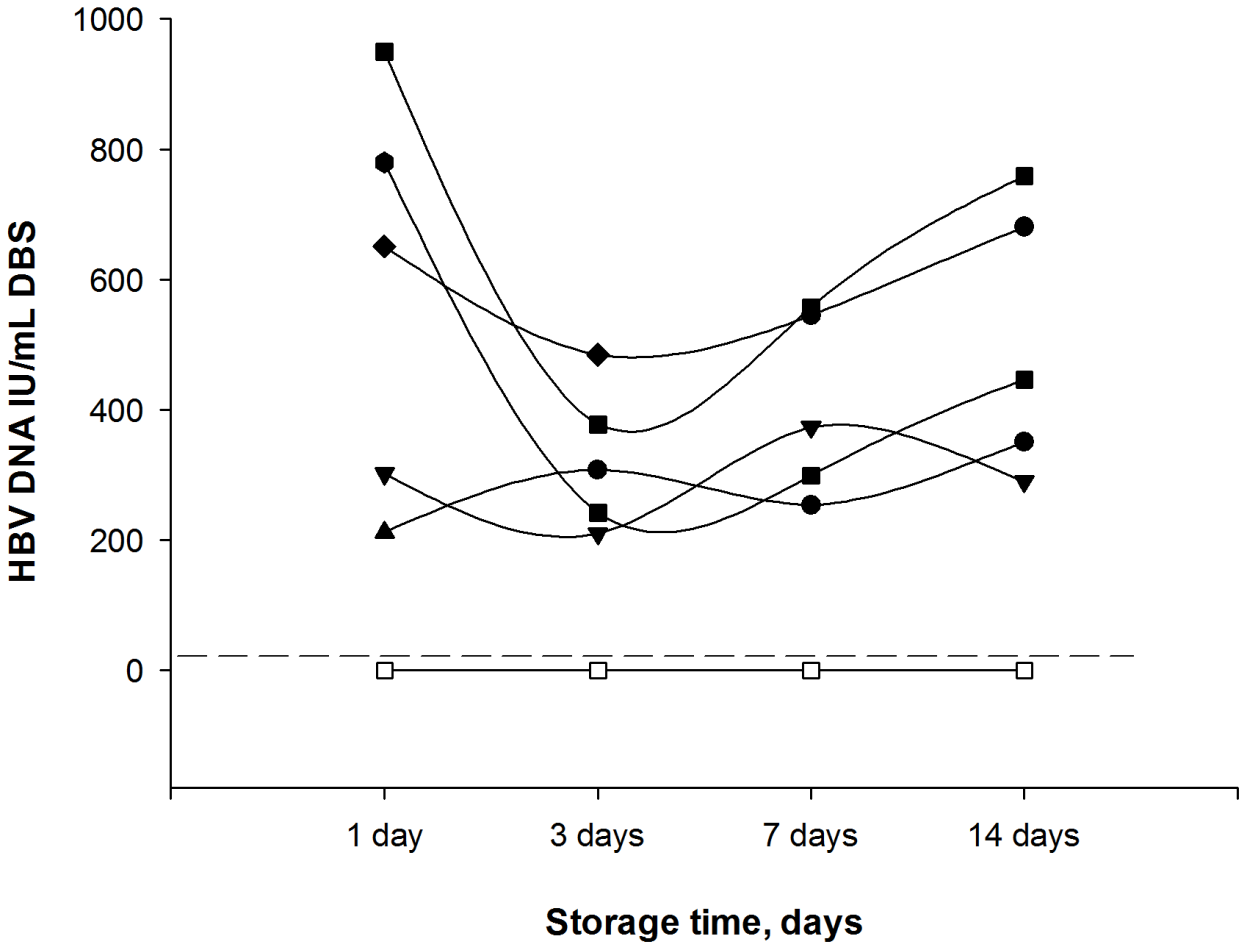
Evaluation of DBS storage relative to HBV DNA titer. DBS samples were tested after storage at room temperature during 1, 3, 7, and 14 days. Each line represents 5 positive samples (black symbols) and 3 negative samples (white symbols). The dotted line represents the positive cut-off value (20 IU/mL).

Serial dilutions of 10 DBS and plasma samples positive for HBV DNA were diluted in negative blood and were run in duplicate to investigate the assay’s capacity to detect low levels of HBV DNA. The limit of HBV DNA detection in DBS was 914.10±157.77 IU/mL (Table 1). HBV DNA was also analyzed after different storage times for five positive patients and three negative patients. In HBV DNA positive samples, a slight decrease was observed for three positive patients at day 3 but there was no significant variation in the titer (IU/mL) (**Figure 4**).

### Quantification of HBV DNA with DBS

HBV DNA values from DBS were also compared with those from plasma. HBV DNA was detected in 100% (50/50) of DBS samples. The HBV DNA viral load in matching plasma samples ranged from 1 215 to 5 987 097 IU/mL. A good correlation was found between DBS and plasma measurements of HBV DNA (R^2^ = 0.86; P<0.0001) (**Figure 3C**). Bland-Altman agreement analysis revealed that DBS specimens were comparable to plasma specimens for measuring DNA HBV values in all samples except one. Notably, the mean difference between plasma and DBS specimens was −0.65 log IU/mL, within the limits of agreement (mean difference ±2SD) (**Figure 3D**).

### Genotype Characterization in Plasma and DBS Samples

HBV genotypes were determined in 10 HBV-infected patients. The HBV DNA viral load in matching plasma samples ranged from 20065 to 5987097 IU/mL. Genotype sequence was obtained in duplicate for all DBS. Full concordance was observed between DBS and matching plasma samples both for genotype and mutations on HBV polymerase gene. One L180M mutation in patient 3 was detected in plasma and DBS (**Table 2**).

**Table 2:** HBV Genotype characterization in DBS and plasma samples.

		Genotype		Mutations	
Samples	Viral load IU/mL	Plasma	DBS	Plasma	DBS
**1**	1.00×10^5^	A	A	wt	wt
**2**	1.04×10^5^	C	C	wt	wt
**3**	1.38×10^5^	A	A	L180M	L180M
**4**	1.64×10^5^	C	C	wt	wt
**5**	2.76×10^5^	D	D	wt	wt
**6**	2.97×10^5^	D	D	wt	wt
**7**	3.50×10^5^	E	E	wt	wt
**8**	3.66×10^5^	B	B	wt	wt
**9**	5.80×10^5^	D	D	wt	wt
**10**	5.99×10^6^	A	A	wt	wt

## Discussion

Our study is the first both to evaluate serological markers and to quantify HBV DNA on DBS using real-time PCR, automated methods, and commercially available assays. Our study demonstrates an excellent sensitivity (98%) and specificity (100%) of DBS for HBsAg and HBV DNA detection.

Recently, DBS have been used to study the prevalence of HBV infection in endemic areas and in specific groups at risk for infection, such as prisoners [19], [21], [22], [23], [24], [25], [26]. However, Forbi et al reported a low sensitivity (78.6%) and specificity (88.6%) for HBsAg detection in DBS compared to serum samples, thus warranting further investigation before DBS testing can be implemented [25]. Villar et al. also used ELISA for HBsAg and anti-HBs detection in DBS samples [26]. For HBsAg, their assay had a sensitivity of 97.62% and specificity of 96.7% and for anti-HBs, sensitivity was 78% and specificity 97.3%.

Chronic replication of HBV is characterized by persistent circulation of HBeAg [4]. In our study, all patient positive for HBeAg in plasma were also positive on DBS.

Regarding HBV DNA detection, a good concordance was observed between the HBV DNA detected in DBS and in plasma. The overall sensitivity with DBS was lower than that for plasma in patients with 1000 IU/mL of HBV DNA, but this might have been improved by extracting DNA from larger DBS samples. Patients should be considered for treatment when HBV DNA levels are above 2000 IU/mL [27]. In our experience, values below 1000 IU/mL of HBV DNA are very uncommon in untreated patients, and therefore this level of sensitivity may be suitable for pretreatment molecular HBV diagnosis. The results of our study indicate that DBS samples represent a promising means for accurately identifying HBV genotype and polymerase gene mutations. DBS could thus increase the applicability of HBV testing, facilitating population-based studies.

Many studies have reported successful genotyping of HIV from DBS and a high genotypic concordance with plasma genotypes. DBS is the preferred specimen type for transmitted HIV-1 drug resistance surveillance when plasma collection is not feasible [28].

Jardi et al reported successful HBV DNA detection on DBS by in-house PCR and nested PCR, in 82 patients [17]. Those authors showed that DBS sensitivity for HBV DNA quantification was about 10^2^ copies/mL. They also detected HBV pre-core mutants (G1896A) in HBeAg-negative chronic hepatitis and evidenced lamivudine resistance of HBV polymerase was evidenced by mutations in the YMDD locus of HBV polymerase**.** But nested PCR cannot generally be recommended for clinical diagnosis due to potential contamination [29]. The originality of our work was to use commercial kits for the extraction and for molecular assays. This approach has several advantages: no expertise is required for assay development and optimization, and quality control can be done using the manufacturer’s control reagents. Moreover, HBsAg and anti-HBs detection was assessed on Whatman FTA DMPK-C cards, which are widely available.

It is important to highlight that the stability and integrity of DNA depend on storage conditions such as temperature, freeze-thaw cycles, and buffer composition [30], [31]. In this study we showed that there was no significant difference in HBsAg, anti-HBs, and HBV DNA levels in DBS at 1, 3, 7, and 14 days of storage at room temperature. By contrast, Tuaillon et al. reported that HCV RNA rapidly degrades in DBS, even when stored at room temperature for less than two weeks [32]. Furthermore, in there HCV-negative samples, absorbance values increased with exposure for about 8 days at room temperature, but these may be false positives. This may limit their use for diagnosis of HCV infection in settings where cold storage is not continuously available [32]. But we used DNA for HBV and Tuaillon et al used RNA for HCV, which is more sensitive to degradation.

Another important issue is that, ideally, sampling should be non-invasive and painless, especially for children and psychiatric groups. Preparation of DNA also needs to be rapid, reliable, and consistent. In any large-scale genetic association project, per-unit cost of DNA preparation becomes high. Also, DNA sample collection is needed for genotyping and pharmacogenetic studies to understand the inter-individual variability of drug responses.

Moreover, collections of DBS are convenient and cost effective alternatives to blood sampling for participants and researchers.

DBS can be mailed out after self-collection, overcoming geographical impediments in resource-limited and endemic areas such as sub-Saharan African countries, who have the highest HBsAg prevalence in the world (up to 12%), and the island nations of the Pacific and the Indian Ocean, where HBsAg is highly endemic (about 10%) [33]. Moreover, DBS can be particularly useful for large-scale screening and improving access to care in regions without molecular biology laboratories. The French National Agency for Research on AIDS and Viral Hepatitis reported that long-term stored DBS showed good accuracy and feasibility for HIV-1 RNA measurements in two African laboratories [34].

DBS cards have been used for detection of antibodies directed against measles virus (MV), for detection of MV RNA, and for MV phylogenetic analysis in epidemiological studies and during MV outbreaks. Furthermore, DBS cards can be used to assess the success rate of vaccination programs by examining seroconversion rates. Ibrahim et al. demonstrated that DBS cards could be used to monitor trends in antibody levels during childhood [35].

DBS is a reliable alternative to serum or plasma collection for HBV testing. We showed that 150 µL of whole blood (three drops) spotted onto filter paper allowed the whole panel of HBV testing: HBsAg, anti-HBs, HBeAg, HBV DNA and genotyping. DBS card sampling using tens of microliters of blood decreases the burden of sampling while opening up new diagnostic possibilities for other virus infections [36].

Tenofovir disoproxil fumarate (TDF) and Entecavir (ETV), two oral antivirals anti HBV drugs, have been approved in the United States and Europe for the treatment of CHB. Recent data reported that in treatment-naive patients, viral resistance to TDF or ETV was not detected after up to 3 years of therapy [37], [38]. One limitation in this study was that the number of HBV mutations was small. This is due to the effectiveness of antiviral agents. Further studies with larger sample sizes should be performed to confirm the results on HBV mutations.

In conclusion, determination of HBV markers in DBS is useful in the screening and management of HBV patients. DBS sampling may improve the access of high-risk populations to diagnosis or monitoring of chronic HBV infection through the measurement of antigen, antibodies, and viral load, allowing clinical application. In addition, the rapid determination of HBV markers allows the doctor to have objective information on patient compliance to treatment.

Part of this study has been presented in an oral presentation in the EASL 2012 symposium (Barcelona, Spain).
